# Effects of Power and Ballistic Training on Table Tennis Players’ Electromyography Changes

**DOI:** 10.3390/ijerph18157735

**Published:** 2021-07-21

**Authors:** Amir Hossein Haghighi, Ali Zaferanieh, Seyed Alireza Hosseini-Kakhak, Ali Maleki, Fabio Esposito, Emiliano Cè, Carlos Castellar, Víctor Toro-Román, Francisco Pradas

**Affiliations:** 1 Faculty of Sport Sciences, Hakim Sabzevari University, Sabzevar 9617976487, Iran; ah.haghighi@hsu.ac.ir (A.H.H.); alizaferanieh@yahoo.com (A.Z.); hosseinik@um.ac.ir (S.A.H.-K.); 2 Department of Biomedical Science for Health, Università degli Studi di Milano, 20122 Milan, Italy; fabio.esposito@unimi.it (F.E.); emiliano.ce@unimi.it (E.C.); 3 Faculty of Sport Sciences, Ferdowsi University of Mashhad, Mashhad 9177948974, Iran; 4 Faculty of Electrical and Computer Engineering, Semnan University, Semnan 3513119111, Iran; amaleki@semnan.ac.ir; 5 ENFYRED Research Group, Faculty of Health and Sports Sciences, University of Zaragoza, 22001 Huesca, Spain; castella@unizar.es (C.C.); franprad@unizar.es (F.P.); 6 School of Sport Sciences, University of Extremadura, Avenida de la Universidad s/n, 10003 Cáceres, Spain

**Keywords:** maximum muscle excitability, root mean square, mean frequency, co-activation

## Abstract

The aim of the present study was to analyze the effects of ballistic and power training on table tennis players’ electromyography (EMG) changes. Thirty male table tennis players, who were able to perform top spin strikes properly, were randomly assigned to three groups: power training (PT; n = 10); ballistic training (BT; n = 10); and no training (CON = control group; n = 10). PT and BT were performed 3 times weekly for 8 weeks. Before and after training programs, a one-repetition maximum test (1RM) and the EMG activity of all the subjects’ upper/lower body muscles while performing top spin strokes were analyzed. After training, significant interactions (group × time) were observed in increasing 1RM strength in upper/lower muscles (*p* < 0.05). However, neither training type had any significant effect on muscle EMG activity. These findings suggest that there should not necessarily be any significant change in the EMG signal after BT and PT despite the increase in muscle strength.

## 1. Introduction

Mr. Ogimura (the former world table tennis champion and president of the International Table Tennis Federation) states that table tennis is ‘*the sport that performs chess while carrying out 100 m running*’. Although it simply expresses table tennis, it is difficult to choose the optimal batting style and to hit a ball that comes flying at irregular timing, spinning, and speed. Table tennis is one of the fastest sports in which the condition for presenting a spectacular game is to have speed of action, speed of reaction, and strong vision of the player. In this field, sometimes, the speed of the ball reaches 200 km per hour. Therefore, table tennis players need high levels of speed, strength, power, flexibility, and quick reactions [1]. In fact, maximum force, speed, acceleration, and power are the main committees in table tennis that allow players to have strong muscle contractions and perform many tasks such as exchanging balls with an opponent [2]. Table tennis is a sport in which change of tactics is required from analyzing a partner’s style. Top-level competition needs high-level performance. Clearly, high-level physical strength is required in the background of the player to develop. The physical strength of a top player who plays actively worldwide is very high and requires intense prolonged play on both sides of the recess for only 1 min [3]. The forehand top spin is one of the most attacking table tennis shots. A table tennis court is relatively small and requires players to respond more quickly than other racket sports [4]. Regarding energy demands in table tennis, anaerobic glycolysis and tri-phosphate-phosphocreatine pathways predominate in very short maximal efforts, along with the aerobic system during recovery [3]. On the other hand, physical qualities such as agility, reaction time, ballistic strength, and coordination are key to performance [5]. Maximal isometric strength and maximal shortening velocity in the force-velocity relationship of muscles are critical variables in table tennis performance. These parameters can be improved through power and ballistic training. It is known that neuromuscular adaptations are specific to different training stimuli [6].

Power is one of the most important characteristics of muscle function and one of the most important capabilities for any athlete in competitive sports [7]. From an exercise perspective, the ability to perform high-intensity to maximum exercises in less than one to a few seconds is called power. According to this subject, any increase in power should be obtained from progress in strength, speed, or a combination of both. Therefore, it can be used from strength training, and especially from the resistance-power training method for this purpose. The main stimulus in these exercises is the dynamics of movement, which is associated with the magnitude of the force exerted in the same effort [8], and it must be performed quickly and explosively to apply the most motor units with the highest contraction rate and create the most neuromuscular compatibility. The main purpose of strength training in high-level competitive sports is the specific progress of players and the relationship between athlete activities and the type of specific strength training that is done in that sport [2].

On the other hand, resistance-ballistic training has been expressed as a special method of gaining optimal speed in some sports [9,10]. Resistance-ballistic training is comparable to throwing because a projectile is released at the end of the range of motion, and therefore, it is possible to design resistance-ballistic training that resembles the movement pattern of table tennis players. Resistance-ballistic training is one of the methods used to overcome the lack of speed in traditional training through full range of motion [11]. These exercises include programs in which the athlete needs to import maximum force in a short period of time with the aim of accelerating an object in the open area (such as jumping, throwing, and kicking). This training method is usually used to increase output power and improve athletic performance. Improvement in performance after resistance-ballistic training has been confirmed in most research literature [8,12].

Increased electrical activity during maximal actions, due to muscle training, could indicate increased activation of the motor units of the trained muscles [13]. An increase in the number of active motor units and/or their firing frequency might result in a higher magnitude of the electromyography (EMG) signal in participants after training [14]. Increased motor unit synchronization may also result in higher EMG amplitudes. Thus, clarifying the effect of both parameters on sport performance could help to design especially suitable strength-training programs for individuals [15]. In this context, myoelectrical activity recordings by means of surface electromyography (EMG) appears a relevant approach to gain a better understanding of the training-induced effect on upper/lower limb muscles’ activity level while performing table tennis strokes. To the best of our knowledge, to date, no studies have quantified the training effect on upper/lower limb muscles’ activity level while performing forehand table tennis top spin strokes.

Of all the strength training modalities, power training (PT) and ballistic training (BT) can help athletes to improve both strength and velocity. Ballistic exercises provide a unique stimulus in which athletes can attempt to accelerate through the full range of motion [16]. BT is comparable to throwing action because a projectile is released at the end of the range and, thus, we can design similar ballistic exercises to table tennis players’ movement patterns. PT is characterized by applying high levels of force in a short period of time and muscle contraction at high speeds [17]. Previous studies have examined joint kinematics [18], joint kinetics [19], and EMG activity [20] during forehand plays. However, information on the design of effective strength training programs for table tennis players is scarce. In this study, EMG data were recorded while making skilled movements, which is a new idea and can open up a new path for other researchers. 

Given that table tennis players perform fast and powerful movements [21], we believe that PT and BT could be interesting for this group. Although training stimuli vary considerably between the two methods, exposure to both types of exercise will provide the neuromuscular adaptation needed to improve strength, power, and speed in such individuals. To develop neuromuscular mechanisms in table tennis, resistance-power and resistance-ballistic training can be effective as a physical process to match the physiological characteristics of nerve and muscle [22]. Physiological adaptations are associated with dynamic resistance-power and resistance-ballistic training programs. These training programs cause mechanical and neuromuscular adaptation in the muscles, which leads to the improvement of muscle function. In addition, the benefits of strength in increasing the speed of action in special exercises are apparently due to the coordinated contractions of the opposing muscles during the movement [21]. Therefore, this study aimed to investigate the effects of 8-week PT and BT on EMG changes of the muscles directly involved in table tennis players’ technical skills (stroke performance). It was hypothesized that the two training modalities would elicit significantly different changes in both muscle excitability and the agonist/antagonist muscle ratio.

## 2. Materials and Methods

The following sections were previously reported by Zaferanieh et al. [23].

### 2.1. Subjects

Thirty men (aged 24 ± 7 years; height 175 ± 6 cm, body weight 74 ± 16 kg) were enrolled in this study. The sample was randomly divided by web page (https://www.randomizer.org/, accessed on 18 March 2019). All were athletes and played at the provincial and national level, had a training seniority of 7 ± 3 years, and attended 5 ± 1 training sessions/week. After being fully informed of the study aims and procedures, they were all provided informed consent and randomly assigned to (i) PT (n = 10), (ii) BT (n = 10), no training (Control, (CON), n = 10). Sample size was calculated in light of the previous research results [24] following a similar approach. The desired sample size was 30 table tennis players capable of performing top spin strokes properly. 

This study was approved by the Ethics Committee of the Hakim Sabzevari University, Iran, with approval ID IR.HSU.REC.1397.015. This committee is responsible for evaluating research with human subjects, which was conducted in accordance with the latest Declaration of Helsinki principles, updated at the World Medical Assembly in Fortaleza (2013). To be enrolled in this study, all the participants underwent a medical examination and had no neuromuscular and orthopedical impairments of upper/lower limbs, and they maintained physical activity, training, and nutritional intake habits during the study. 

### 2.2. Experimental Design

The present research work was designed as a parallel three-group longitudinal study. During the first sessions, the participants became familiarized with one repetition maximum test: maximum muscle excitation with measurements during the stroke test. The maximum muscle excitation test was recorded for each investigated muscle. For reliability purposes, during the second session, the same measurements were taken again for further analyses. Each session was interspersed by at least 2 days. Thereafter, 8-week training protocols (PT or BT) were administered. The same measurements as in the second session were retaken within 24 h since training ended. CON performed the programmed table tennis training only.

### 2.3. Measurements

Anthropometrics and fat mass: Anthropometric characteristics were assessed early in the day in the fasting condition. Body height was measured to the nearest 0.1 cm with a wall-mounted stadiometer (Seca 220, Hamburg, Germany). Body weight was measured to the nearest 0.01 kg on calibrated electronic digital scales (Seca 769, Hamburg, Germany) in the nude and barefoot. A Holtain© 610ND (Holtain, Crymych, Wales, UK) skinfold compass (accurate to ±0.2 mm) was used for the fat mass assessments. Height, weight, and three skinfolds (chest, abdomen, and thigh) were obtained for further analysis. The percentage of fat mass (FM) was determined following the guidelines of Jackson and Pollock [25]. All measurements were performed by the same operator according to the recommendations of the International Society for the Advancement of Kinanthropometry [26].

1RM determination. The chest press test and the leg extension test were selected to perform the one-repetition maximum (1RM) test. 1RM was determined following the guidelines of Kraemer et al. [7]. The following procedures were followed during the test protocol: (i) a warm-up including 5–10 repetitions at 40–60% of the estimated 1RM, (ii) 1 min to rest with light stretching followed by 3–5 repetitions at 60–80% of the estimated 1RM, and (iii) three to five attempts to reach the 1RM with a 5 min rest interval between each new lift. The successfully lifted maximum load was identified as 1RM [27]. All the participants were familiar with both exercises.

Maximum muscle excitation test. The maximum muscle excitation of deltoid anterior (DA), deltoid posterior (DP), biceps *brachii* (BB), triceps *brachii* (TB), flexor *digitorum* (FD), extensor *digitorum* (ED), biceps *femoris* (BF), and rectus *femoris* (RF) muscles of the dominant limb (corresponding to the racket side) were determined under isometric conditions. For DA and DP, the participants were instructed to flex or extend their elbow to 90° so that their hand pointed upward. Then, they were asked to make a closed fist with the hand of the flexed arm and provide maximal force to produce shoulder flexion against manual resistance [28]. For both TB and BB, the participants were instructed to flex or extend their elbow at 90°. Then, they applied maximal force in an attempt to extend their elbow against manual resistance [29]. For FD and ED, their elbow flexed at 90° was held, and the metacarpus extended or flexed against manual resistance. For BF and RF, the participants sat on a leg extension machine with their hip flexed at 90° and their knee at 60° (considering full knee extension = 0°) [30]. Then, they applied maximal force by flexing either their ankle against manual resistance or their knee against the machine’s ankle support. The duration of each attempt was 5 s. Three attempts were completed for each movement and were interspersed by 3 min of recovery. The operators gave standardized verbal encouragements. The maintenance of the joint angles required during tests was monitored by two bi-axial electrogoniometers (mod. TSD 130A; Biopac System, Santa Barbara, CA, USA).

Stroke test. After a 30-min rest from the maximum muscle excitation test, the participants performed the stroke test, which consisted of repeating 10 consecutive strokes. During this test, the muscle excitation level of the eight muscles tested during the maximal muscle excitation test was detected. The participants performed 10 consecutive strikes by applying the most frequently used modality by players, i.e., forehand spin [31]. A table tennis robot (Robo-Pong 2040 DonicR, Volkingen, Germany) was used to ensure high ball bounce reproducibility and ball velocity, even in the event of any possible minor variation in ball speed and/or ball frequency. All the participants used their own racket. Stroke series were invalidated when at least one-third of trials was missed or if a series of three consecutive errors occurred, in which case a new test was conducted after 48 h.

### 2.4. Training Program

The exercises of the training program were performed during three sessions per week for 8 weeks. The participants performed warm-up (15 min) and cool-down (15 min of general mobility and stretching) exercises before and after the main training activities. Experienced instructors supervised training activities. The training program was similar to that reported by Zaferanieh et al. [23]. Training programs are shown in Table 1 and Table 2.

For PT, the subjects performed exercises at six stations. Each exercising session involved performing 1 h of resistance-power exercises. Sessions started with general mobility exercises, running, and the following non weight-bearing exercises. The time between each set and the next set was 1–2 min, and it was 3–5 min from one station to the next. The training stations for the resistance-PT group included three movements for the lower trunk, leg press, leg extensions, lying leg curls, and three movements for the upper trunk, Barbell curl, machine shoulder (military) press, and bench press (Table 1).

Ballistic training was also composed of six stations. Each session started with a 15-min warm up, including running, and three sets of five repetitive squat jumps without weight and jumping up and forth without weight. A 2-min between-set recovery and a 4-min between-station rest were included. A 3 kg medicine ball was used [23] (Table 2).

### 2.5. Surface EMG Recording

The sEMG signal was detected during the maximum muscle excitation and stroke test. The skin area under sEMG electrodes was shaved, cleaned with ethyl alcohol, gently abraded with fine sand paper, and prepared with a conductive cream (Nuprep^®^, Weaver and Co., Aurora, CO, USA) to obtain an interelectrode impedance below 2000 Ω. The sEMG signal was detected by two Ag/AgCl rounded electrodes with solid hydrogel (mod H124SG Kendall ARBO; diameter: 10 mm; interelectrode distance: 20 mm; Kendall, Donau, Germany) following the European Recommendations for Surface Electromyography [32]. The sEMG electrodes for DA were placed on the mid-belly muscle approximately 4 cm below the clavicle [29]. The sEMG electrodes for DP were placed on the mid-belly muscle. The sEMG electrodes for BB and TB were placed on the mid-belly of the long head midway between the acromion process of the scapula and the olecranon process of the ulna [33]. The sEMG electrodes for FD, ED, RF, and BF were placed on mid-belly muscles. A ground electrode was placed on the seventh cervical spinous process. Electrodes were equipped with a probe (probe mass: 8.5 g, BTS Inc., Milan, Italy), allowing the detection and transfer of the sEMG signal wirelessly. The sEMG signal was acquired at 1000 Hz, amplified (gain: 2000, impedance and the common rejection mode ratio of the equipment were > 10^15^ Ω//0.2 pF and 60/10 Hz 92 dB, respectively), and exported to a wireless electromyographic system (FREEEMG 300, BTS Inc., Milan, Italy) that digitized (1000 Hz) and filtered (band-pass 10–500 Hz) the raw sEMG signals.

### 2.6. Data Analysis

The sEMG signals from both the maximum muscle excitation and stroke tests were analyzed in time and frequency domains. For maximum muscle excitation, the averages of both the root mean square (RMS) and the mean frequency (MF) corresponding to the central 2 s were considered. During the stroke test, muscle activation was considered when the EMG signal was > 3 standard deviations over the baseline signal noise (AcqKwnoledge 5.1, Biopac System, Santa Barbara, CA, USA). From these time windows, RMS and MF were calculated and averaged over the central 100 ms of each stroke. The RMS and MF values from strokes were compared to check for any possible fatiguing effect (see the Section 2.7). Then, EMG RMS and MF during each stroke were averaged [34] and stored in a laptop for the data analysis. Finally, as recommended in a previous research work [35], the co-activation of the knee agonist/antagonist muscle pairs (CoAct) was computed from the EMG signal at each maximal voluntary contraction level [36]:

CoAct = 2 · EMG_ANTAGO_/(EMG_AGO_ + EMG_ANTAGO_) · 100%.

EMG_ANTAGO_: antagonist electromyography; EMG_AGO_: agonist electromyography

The pair of agonist/antagonist muscles was chosen due to the forehand top spine strike, respectively: (i) DA/DP, (ii) BB/TB, (iii) FD/ED, and (iv) BF/RF.

### 2.7. Statistical Analysis

The Shapiro–Wilk test was used to check the normality of variables. The intraclass correlation coefficient (ICC) and the standard error of measurement percentage change (SEM%) was calculated to determine the inter-session reliability of the EMG parameters. The ICC was interpreted as follows: >0.90: very high; 0.89–0.70: high; 0.69–0.50: moderate [37]. The minimal detectable change at the 95% confidence interval (MDC_95%_) was used to detect the sensitivity of the effects on the EMG parameters between pre- and post-trainings [38]. The EMG data over 10 forehand strokes (pre and post) were recorded, and their mean was calculated. A two-way ANOVA (group × time) was applied to check for any possible differences in the RMS, MF, and CoAct values obtained during the stroke test. A post hoc test was applied with Bonferroni’s correction. Statistical significance was set at *p* < 0.05. Cohen’s d with a 95% confidence interval was provided for each comparison purposes. Cohen’s d effect size was calculated and interpreted as follows: 0.00–0.19: trivial; 0.20–0.59: small; 0.60–1.19: moderate; 1.20–1.99: large; ≥2.00: very large [39]. Data were presented as mean ± standard deviation (SD). The statistical analysis was performed by a statistical software package (IBM SPSS Statistics v. 25, Armonk, NY, USA).

## 3. Results

Study compliance. Attendance at training sessions was 92% (22/24 training sessions). Three participants dropped from the study (two participants from PT and one participant from BT).

Reliability. The inter-session reliability for EMG RMS and EMG MF during the maximum muscle excitability and stroke tests was measured. The ICC and SEM% for EMG RMS ranged from 0.899 and 3% to 0.987 and 14%, respectively. The ICC and SEM% for EMG MF went from 0.921 and 2% to 0.991 and 5%. For all the parameters, MDC_95%_ was between 5% and 38%.

Body mass index, fat mass, and 1RM. The BMI, FM, and the 1RM results for both chest press and leg extension in the three study groups and for pre- and post-intervention are presented in Table 3. Increases in 1RM after PT and BT were noted compared to CON (*p* < 0.05).

Maximum muscle excitation. The differences in EMG RMS and MF obtained during the maximum muscle excitation tests for all the muscles are reported in Table 4 and Table 5.

In Table 4, the ANOVA disclosed a significant time × intervention interaction EMG RMS for BF (*p* = 0.024). A significant increment in EMG RMS was found in BF in CON (*p* < 0.05) and after PT (*p* < 0.05). Moreover, an increase in DA for CON took place (*p* < 0.05). 

In Table 5, the ANOVA disclosed a significant time × intervention interaction EMG MF for RF (*p* = 0.011) and BF (*p* = 0.035). Table 5 shows a decrease of EMG MF in BF after PT (*p* < 0.05) and for CON in RF (*p* < 0.05).

Stroke test. Table 6 and Table 7 showed differences in EMG RMS and MF obtained during the stroke test in all muscles. 

In Table 6, no significant time × intervention interactions were recorded. EMG RMS decreased significantly in RF after PT (*p* < 0.05) and for ED in CON (*p* < 0.05). Moreover, EMG RMS decreased in BF in CON (*p* < 0.05).

Table 7 shows increase of EMG MF in BB and ED for CON (*p* < 0.05). However, EMG MF significantly decreased in TB after BT (*p* < 0.05). 

Agonist/antagonist ratio. The changes in the agonist/antagonist muscle ratio for muscle excitation during the post- intervention stroke test are presented in Table 8. 

The ANOVA indicated a significant time × intervention interaction in RF/BF (*p* = 0.031). The main effects for time appeared in FD/ED and RF/BF (*p* < 0.05). RF/BF significantly reduced after PT and in CON (*p* < 0.05), but a significant increase in FD/ED was observed in CON (*p* < 0.05).

## 4. Discussion

To the best of our knowledge, this is the first study to analyze the effects of BT and PT on table tennis players’ EMG changes. The main findings of our study were obtained when performing BT and PT, and they were effective for increasing 1RM strength in both upper/lower muscles. However, only PT demonstrated some sporadic increments in the maximum excitability level of muscles, some adjustment in muscle excitability during strokes, and also in the agonist/antagonist ratio for lower limb muscles during stroke execution. These findings suggest that BT and PT are unable to clearly improve table tennis player’s muscle excitability level and agonist/antagonist coordination.

### 4.1. Maximum Muscle Excitability

In the present study, BT was not effective in EMG RMS and MF during the maximum muscle excitability of all muscles, and only PT was effective in EMG RMS and MF in BF. BT was not effective in EMG RMS and MF during stroke in all muscles, and only PT was effective in EMG RMS in RF. BT was not effective in EMG RMS and MF for the agonist/antagonist muscle ratio for all muscles and only PT was effective in EMG RMS in RF/BF. In a study by Pincivero et al. [40], they showed that strength training with maximum muscle contractions at different intensities leads to muscle changes. They also showed that maximal contractions in 30 healthy male and female subjects (without any strength training for 6 months) with intensities of 10 to 90% of maximum muscle contraction increased the mean frequency of muscle power. Significantly, there is a further increase in the average frequency of muscle power at higher intensities than lower intensities, so that the highest values of mean frequency of muscle strength were obtained at 90% of the maximum muscle voluntary contraction. In other words, the recruitment and evacuation of more motor units, higher synchronization of motor units, higher coding rate of these units [41], speed of guiding muscle fibers, and to some extent firing frequency of more motor units [42] and increase in the extent of strength development after strength training [43] are obtained at this intensity. Amarantini et al. [44] also concluded that with increasing intensity of resistance training, the average frequency of muscle strength increases more. Agaard et al. [45] showed that heavy resistance training for 14 weeks reduced the average power frequency only in the vastus lateralis muscle. The type of exercise in this group included 10–15 maximum repetitions in the first 5 sessions and 6–10 maximum repetitions in the rest of the exercises. In the last 4 weeks, these exercises were performed with 6–8 maximum repetitions. The team the researcher was working with was untrained. In a way, non-athletes acquire different neuromuscular adaptations to athletes as a result of strength training, and our expectations for non-athlete training adaptations vary from athlete to athlete. Athletes usually adapt faster than non-athletes due to their high physical fitness. In addition, Behm and St-Pierre [46] observed a slight decrease in muscle activation and suggested that short-duration contractions (e.g., forehand top spine strikes) did not necessarily lead to significant changes in EMG activity compared to longer duration contractions. A lack of significant EMG signal changes may have been influenced by the monitored muscles’ fiber-type composition and relative muscle involvement while exercising.

In order to interpret frequency changes, it is necessary to be aware that the EMG spectrum is determined by the shapes of motor unit action potentials (MUAPs) generated along muscles. In turn, MUAP translates a shape into a time (MUAPt), as observed by the measuring device at a fixed position. The translation from MUAPs into MUAPt results from conduction velocity (CV) [47] while table tennis players perform, for example, top spine strikes. An interference EMG is generated by different overlapping MUAPs. The training type may change many muscle properties, especially those that change the size, fiber composition, and neuromuscular control of muscles [48]. However, other discussions have more frequently expressed changes in CV that then alter the mean frequency (MF) of spectra. CV is influenced by the type [49], proportion [50], and cross-sectional area of muscle fibers, among other factors. However, Troni et al. [51] showed that CVs were almost normally distributed and formed a single peak. Hence, there is a need to consider that fiber type, proportion, and cross-sectional area also change MUAP shape and, thus, spectra. Farina et al. [49] indicated several reasons for changing the MF of EMG spectra. A study on the abductor pollicis brevis muscle revealed that besides changes in CVs, spectra shape also altered, which indicates changes in MUAP shape. One of the factors that markedly determines MUAP shape and size is the endplate distribution in the innervation zone. The innervation zone [52] represents a primary component that affects the MUAP shape and, thus, spectra. Muscle characteristics may change after a period of training. Therefore, it is possible that the spectra of ballistics trained and power trained athletes may take different forms. As our results in the present study did not show any significant changes after BT and PT in MF, it would seem that these results differed because of the distinct signal record type (during top spine strokes). The isometric EMG measurements published by several researchers [53] have been unable to explain the spectral changes that we observed while dynamic movements were performed (e.g., table tennis players’ movements). It is known that different dynamic movements could generate modifications in the muscle. Shepstone et al. [54] measured larger diameters in muscle fibers after fast strength training for both fibers type. According to the classic EMG theory, a larger fiber diameter should lead to higher CV, and shifts to higher frequencies were experimentally confirmed [55]. Based on these two relations, an increase in both fiber diameter for sprint-trained athletes (e.g., table tennis player) and CV, sprint-trained participants would be expected to have a higher MF for their spectra.

However, our results did not show any change to MF after BT and PT with our table tennis players. According to these results, we conclude that the usual explanation of a relation among fiber type, CV, and frequency would not be sufficiently understood to explain the obtained results. The results herein presented are in accordance with those studies reporting that even though 1RM changed, the MF did not. In some cases, the MF was independent of fiber diameter. We could argue that basic relations are usually measured under non dynamic loading conditions. Therefore, perhaps table tennis players have an increased ability to synchronize MUAP, which is why no changes in the MF were seen, in which circumstances synchronization increases are not known and should be considered to explain the effect.

The shape of the spectra depends on the recruitment of the selected MU (motor units) (central control) and on the peripheral buildup of the interference EMG. A high correlation of MU can also be described as synchronization [56]. Semmler and Nordstrom [57] were able to show that strength-trained subjects had a greater degree of synchronization of MU than untrained subjects. The higher the synchronization of MUs, the more the muscle fibers became activated. This helped in performing a fast force production, such as that needed in table tennis players, where muscles have to work in concert. The synchronization of MU leads to an absolute increase of power in the EMG spectra at lower frequencies and to a relative decrease of power at high frequencies. Synchronization results in a downshift of the MF [56]. These findings are in contrast to the increase in MF during isometric contractions observed for many years [47]. It is often supposed that during isometric contractions performed at maximum voluntary contraction, all muscle fibers will be activated. However, in dynamic conditions, spectral changes may result from specifically selected muscle fibers during the actual movement of the performing athlete. The selected muscle fibers strongly depend on their availability. Muscle biopsies indicate which fibers are available and how the proportion changes over the training period. They do not reflect the immediate selection of fibers. Although we may not be able to explain and fully understand the details behind the spectra, this work clearly shows that the spectra change in a systematic way and therefore contain practical reliable information about the training of the athlete. Therefore, an EMG spectrum recorded during a dynamic sports movement yields its own information about the muscle condition in addition to the information obtained by EMG spectra recorded for isometric contractions or muscle biopsies.

### 4.2. Agonist/Antagonist Ratio

There was no significant difference between the groups in the agonist/antagonist muscles coactivation ratio index in any of the muscle pairs. The activity of agonist and antagonist muscles has been extensively studied in various studies. For example, Amarantini et al. [44] concluded that resistance-strength training at different intensities leads to adaptations in the vastus lateralis and vastus medialis muscles relative to the two semitendinosus and biceps femoris muscles; the result is a reduction in co-activation of these two opposite muscles. The decrease in co-activation of the two opposite muscles means that the role of the agonist muscles in producing maximum strength and power has increased and the antagonist muscles have decreased. An important reason for this decrease can be considered the role of supraspinal and corticospinal mechanisms [43]. In other words, a type of neuromuscular adaptation due to the increase in muscle strength following strength training leads to a change in the motor cortex of the brain. The increasing co-activation of antagonists due to strength training also refers to the same subject. Increased antagonistic muscle coactivation means that the stimulation of Renshaw or inhibitory cells in the cerebral cortex and the stimulation of inhibitory Purkinje cells in the cerebellum are increased, leading to an increased inhibitory role of antagonist muscles in affecting the cerebral cortex through Renshaw cells [58]. Aagaard et al. [43] reported an increase in H reflex and V wave responses following strength training as a sign of an increase in corticospinal nerve excitation and increased excitability of motor neurons. Hakkinen et al. [59] also concluded that 6 months of resistance training on two groups of middle-aged men and women reduced co-activation in the quadriceps femoris and hamstrings muscles (especially biceps femoris muscle). Stock et al. [60] also found that increases in EMG amplitude occurred during 10 weeks of resistance training for the rectus femoris and quadriceps femoris muscles in agonists for women but not for men.

Co-activation refers to the concurrent activation of agonist and antagonist muscles. A triphasic pattern of EMG activity whereby a marked burst of agonist activity is followed by a shorter “braking” burst from the antagonistic musculature, and by finally a second agonist burst, during rapid or ballistic contractions, has been suggested. Performing movement may be partially contingent on this activity’s net effect. Baker and Newton [61] suggested that an increase during bench press throwing performance after antagonist musculature loading via a set of eight ballistic bench pulls could result from an alteration to the triphasic pattern. They postulated that a shorter braking phase from the antagonist would allow a longer initial burst from the agonist and would, therefore, allow a better performance measure [48,62]. No augmentation in the EMG data was herein observed, which could be due to the upper muscles modality of a top spin strike performed at high speed compared to the intervention applied by Baker and Newton [61], which involved eight ballistic bench pulls. However, there were significant differences in some muscles compared to the pretest. However, these changes were not enough to see the differences between the groups. However, when all the muscles are considered, it can be concluded that in this study, the two types of PT and BT training did not change the EMG signal. The EMG data showed significant changes in the lower muscles’ agonist/antagonist ratio, which was probably due to slower foot speed during top spin strikes. Stored elastic energy and stretch reflex may play a role in movements involving antagonist loading performed immediately before agonist activity [63]. Nevertheless, given the type of the movements during top spin strokes, and the combination of no ballistic and ballistic movements applied, both stored elastic energy and stretch reflex were not considered factors. Our study has some limitations: (a) it does not control subjects’ nutrition; (b) it does not assess the motivation and mental states, and individual differences, while exercising and performing tests; (c) it does not control individuals’ daily activities while our research was underway; and (d) there was no valid method for recording and analyzing the EMG signal for tennis players’ kicks.

## 5. Conclusions

BT and PT showed no change in EMG in table tennis players. However, BT and PT produced a significant increase in 1RM. It is necessary to define a valid method to record and analyze EMG data in particular. The research literature on this topic is generally very limited, and more research is needed in this area.

## Figures and Tables

**Table 1:** PT program.

Week	1	2	3	4	5	6	7	8
Load (%1RM)	60–65	60–65	65–70	65–70	70–75	70–75	75–80	75–80
Repetition × sets	(10–12) ∗ 3	(10–12) ∗ 3	(8–10) ∗ 3	(8–10) ∗ 3	(6–8) ∗ 3	(6–8) ∗ 3	(4–6) ∗ 3	(4–6) ∗ 3
Stations	6	6	6	6	6	6	6	6

1RM: one-repetition maximum.

**Table 2:** BT program. Data are presented as repetitions × set.

Week	1	2	3	4	5	6	7	8
Throw medicine ball on chest	8 ∗ 3	8 ∗ 3	10 ∗ 3	10 ∗ 3	10 ∗ 4	10 ∗ 4	12 ∗ 4	12 ∗ 4
Knee tuck jump with weight (3 kg)	8 ∗ 3	8 ∗ 3	10 ∗ 3	10 ∗ 3	10 ∗ 4	10 ∗ 4	12 ∗ 4	12 ∗ 4
Swimming launch	8 ∗ 3	8 ∗ 3	10 ∗ 3	10 ∗ 3	10 ∗ 4	10 ∗ 4	12 ∗ 4	12 ∗ 4
Throw medicine ball on the right side	20 ∗ 3	20 ∗ 3	30 ∗ 3	30 ∗ 3	30 ∗ 4	30 ∗ 4	30 ∗ 4	30 ∗ 4
Throw medicine ball on the left side	20 ∗ 3	20 ∗ 3	30 ∗ 3	30 ∗ 3	30 ∗ 4	30 ∗ 4	30 ∗ 4	30 ∗ 4

**Table 3:** Changes in BMI, FM%, and muscle strength after the 8-week intervention.

Physical Fitness and Skill		Group	Pre (m ± SD)	Post (m ± SD)	*p*	*F*	*d* (CI_95%_)
BMI (kg/m^2^)		PT (n = 8)	23.8 ± 3.1	23.3 ± 2.9	0.176	1.854	0.29
		BT (n = 9)	24.7 ± 5.9	24.6 ± 5.4			0.03
		CON (n = 10)	23.9 ± 4.0	23.9 ± 3.6			0.01
FM (%)		PT (n = 8)	12.0 ± 6.0	10.3 ± 5.0 *	0.082	2.747	0.38
		BT (n = 9)	11.2 ± 8.0	10.2 ± 9.0			0.12
		CON (n = 10)	11.5 ± 6.0	11.8 ± 7.0			0.13
Muscle strength (kg)	Chest press	PT (n = 8)	73.0 ± 31.0	83.0 ± 34.0 *^,#^	0.006	6.305	0.56
		BT (n = 9)	64.0 ± 13.0	70.0 ± 12.0 *			0.76
		CON (n = 10)	63.0 ± 13.0	63.0 ± 12.0			0.01
	Leg extension	PT (n = 8)	58.0 ± 31.0	81.0 ± 32.0 *^,#^	0.001	8.362	0.64
		BT (n = 9)	54.0 ± 17.0	67.0 ± 23.0 *			0.65
		CON (n = 10)	59.0 ± 15.0	61.0 ± 16.0			0.23

BMI: body mass index; FM: fat mass; PT: power training group, BT: ballistic training group, CON: control group; * *p* < 0.05 vs. Pre; # *p*< 0.05 vs. CON.

**Table 4:** Changes in the EMG root mean square (RMS) in each muscle during the maximum muscle excitability test after the 8-week intervention.

	EMG RMS (mV)					
	Group	Pre (m ± SD)	Post (m ± SD)	*p*	*F*	*d* (CI_95%_)
DA	PT	0.844 ± 0.209	0.897 ± 0.188	0.214	1.652	0.085
	BT	0.715 ± 20.92	0.718 ± 0.250			0.001
	CON	0.676 ± 236	0.847 ± 0.245 *			0.376
DP	PT	0.745 ± 173	0.749 ± 0.209	0.267	0.768	0.001
	BT	0.609 ± 307	0.550 ± 0.202			0.074
	CON	0.641 ± 208	0.565 ± 0.228			0.108
BB	PT	0.917 ± 0. 237	0.936 ± 0.126	0.252	1.466	0.020
	BT	0.825 ± 218	0.779 ± 0.240			0.179
	CON	0.851 ± 0.121	0.895 ± 0.181			0.156
TB	PT	0.877 ± 0.161	0.915 ± 0.175	0.678	0.395	0.047
	BT	0.753 ± 0.292	0.721 ± 0.237			0.044
	CON	0.772 ± 0.230	0.789 ± 0.204			0.013
FD	PT	0.878 ± 0.159	0.1003 ± 0.085	0.192	1.777	0.404
	BT	0.875 ± 0.140	0.862 ± 0.131			0.017
	CON	0.774 ± 0.200	0.883 ± 0.187			0.236
ED	PT	0.750 ± 0.199	0.898 ± 0.169	0.200	1.726	0.235
	BT	0.769 ± 0.231	0.776 ± 0.205			0.001
	CON	0.787 ± 0.223	0.739 ± 0.158			0.091
RF	PT	0.866 ± 0.464	0.751 ± 0.415	0.850	0.163	0.170
	BT	0.717 ± 0.313	0.668 ± 0.271			0.020
	CON	0.764 ± 0.319	0.779 ± 398			0.001
BF	PT	0.633 ± 0.351	0.660 ± 0.313 *	0.024	4.385	0.854
	BT	0.565 ± 0.222	0.818 ± 0.427			0.387
	CON	0.490 ± 179	0.570 ± 0.147 *			0.824

RMS: root mean square; EMG: electromyography; DA: deltoid anterior; DP: deltoid posterior; BB: biceps brachii; TB: triceps brachii; FD: flexor digitorum; ED: extensor digitorum; RF: rectus femoris; BF: biceps femoris. * *p* < 0.05 vs. Pre.

**Table 5:** Changes in the EMG mean frequency (MF) in each muscle during the maximum muscle excitability test after the 8-week intervention.

	EMG MF (Hz)					
	Group	Pre (m ± SD)	Post (m ± SD)	*p*	*F*	*d* (CI_95%_)
DA	PT	125 ± 12	122 ± 18	0.810	0.212	0.052
	BT	125 ± 15	126 ± 19			0.009
	CON	122 ± 16	120 ± 18			0.021
DP	PT	133 ± 9	116 ± 16	0.099	2.566	0.479
	BT	127 ± 16	122 ± 12			0.108
	CON	120 ± 13	123 ± 12			0.036
BB	PT	140 ± 18	120 ± 24	0.419	0.902	0.437
	BT	115 ± 15	109 ± 13			0.167
	CON	120 ± 24	117 ± 22			0.009
TB	PT	143 ± 15	142 ± 24	0.781	0.250	0.008
	BT	134 ± 19	127 ± 17			0.112
	CON	134 ± 14	126 ± 25			0.126
FD	PT	123 ± 18	143 ± 30	0.562	0.591	0.282
	BT	131 ± 19	135 ± 21			0.053
	CON	131 ± 29	137 ± 15			0.029
ED	PT	163 ± 16	152 ± 19	0.119	2.334	0.245
	BT	151 ± 17	156 ± 8			0.070
	CON	162 ± 24	145 ± 11			0.324
RF	PT	125 ± 7	127 ± 19	0.011	5.574	0.022
	BT	122 ± 10	127 ± 11			0.233
	CON	127 ± 13	104 ± 25 *			0.441
BF	PT	161 ± 13	121 ± 9 *	0.035	3.877	0.915
	BT	151 ± 20	141 ± 17			0.275
	CON	141 ± 23	126 ± 16			0.237

MF: mean frequency; EMG: electromyography; DA: deltoid anterior; DP: deltoid posterior; BB: biceps brachii; TB: triceps brachii; FD: flexor digitorum; ED: extensor digitorum; RF: rectus femoris; BF: biceps femoris. * *p* < 0.05 vs. Pre.

**Table 6:** Changes in the EMG root mean square (RMS) in each muscle during the stroke test after the 8-week intervention.

	EMG RMS (mV)					
	Group	Pre (m ± SD)	Post (m ± SD)	*p*	*F*	*d* (CI_95%_)
DA	PT	0.637 ± 0.281	0.647 ± 0.277	0.311	1.231	0.002
	BT	0.401 ± 0.231	0.391 ± 0.259			0.003
	CON	0.444 ± 0.204	0.598 ± 0.318			0.283
DP	PT	0.425 ± 0.230	0.342 ± 0.156	0.358	1.075	0.213
	BT	0.329 ± 0.218	0.251 ± 0.106			0.157
	CON	0.295 ± 0.185	0.317 ± 0.126			0.022
BB	PT	0.550 ± 0.230	0.603 ± 0.335	0.390	0.982	0.086
	BT	0.483 ± 0.287	0.419 ± 0.221			0.088
	CON	0.578 ± 143	0.632 ± 0.175			0.075
TB	PT	0.313 ± 0.115	0.264 ± 0.110	0.189	1.793	0.277
	BT	0.256 ± 0.131	0.220 ± 0.104			0.136
	CON	0.293 ± 0.147	0.357 ± 0.196			0.111
FD	PT	0.671 ± 0.290	0.790 ± 0.176	0.253	1.462	0.386
	BT	0.592 ± 0.224	0.533 ± 0.184			0.246
	CON	0.573 ± 0.249	0.676 ± 0.208			0.090
ED	PT	0.539 ± 0.240	0.470 ± 0.201	0.566	0.584	0.121
	BT	0.593 ± 0.278	0.393 ± 0.162			0.323
	CON	0.548 ± 0.243	0.402 ± 0.181 *			0.372
RF	PT	0.420 ± 0.184	0.215 ± 0.146 *	0.059	3.207	0.580
	BT	0.318 ± 0.164	0.335 ± 0.155			0.007
	CON	0.362 ± 0.152	0.367 ± 0.164			0.001
BF	PT	0.314 ± 0.094	0.254 ± 0.140	0.678	0.395	0.466
	BT	0.281 ± 0.076	0.401 ± 0.264			0.254
	CON	0.245 ± 0.047	0.218± 0.078 *			0.659

RMS: root mean square; EMG: electromyography; DA: deltoid anterior; DP: deltoid posterior; BB: biceps brachii; TB: triceps brachii; FD: flexor digitorum; ED: extensor digitorum; RF: rectus femoris; BF: biceps femoris; * *p* < 0.05 vs. Pre.

**Table 7:** Changes in the EMG mean frequency (MF) in each muscle during the stroke test after the 8-week intervention.

	EMG MF (Hz)					
	Group	Pre (m ± SD)	Post (m ± SD)	*p*	*F*	*d* (CI_95%_)
DA	PT	114 ± 17	123 ± 24	0.085	2.756	0.195
	BT	119 ± 23	110 ± 20			0.288
	CON	108 ± 20	115 ± 19			0.137
DP	PT	115 ± 15	120 ± 18	0.123	2.302	0.121
	BT	119 ± 18	110 ± 18			0.366
	CON	109 ± 9	110 ± 18			0.011
BB	PT	101 ± 24	103 ± 18	0.475	0.768	0.010
	BT	93 ± 19	94 ± 7			0.001
	CON	94 ± 11	102 ± 16 *			0.410
TB	PT	117 ± 17	115 ± 14	0.424	0.891	0.068
	BT	113 ± 17	106 ± 16 *			0.454
	CON	109 ± 12	107 ± 15			0.059
FD	PT	109 ± 20	131 ± 17	0.488	0.741	0.443
	BT	111 ± 16	118 ± 13			0.132
	CON	111 ± 21	125 ± 14			0.296
ED	PT	157 ± 24	149 ± 16	0.551	0.613	0.079
	BT	166 ± 23	150 ± 13			0.258
	CON	161 ± 24	138 ± 9 *			0.504
RF	PT	115 ± 12	118 ± 6	0.211	1.664	0.088
	BT	108 ± 15	115 ± 15			0.097
	CON	102 ± 12	93 ± 16			0.164
BF	PT	118 ± 21	113 ± 16	0.359	1.071	0.055
	BT	113 ± 24	116 ± 17			0.056
	CON	94 ± 18	104 ± 13			0.176

MF: mean frequency; EMG: electromyography; DA: deltoid anterior; DP: deltoid posterior; BB: biceps brachii; TB: triceps brachii; FD: flexor digitorum ioradialis; ED: extensor digitorum; RF: rectus femoris; BF: biceps femoris. * *p* < 0.05 vs. Pre.

**Table 8:** Changes in the agonist/antagonist muscle ratio during the stroke test after the 8-week intervention.

	EMG RMS (%)					
	Group	Pre (m ± SD)	Post (m ± SD)	*p*	*F*	*d* (CI_95%_)
DA/DP	PT	121 ± 20	132 ± 26	0.758	0.281	0.231
	BT	109 ± 28	115 ± 28			0.212
	CON	122 ± 21	127 ± 15			0.111
BB/TB	PT	125 ± 20	132 ± 22	0.460	0.804	0.294
	BT	127 ± 15	129 ± 16			0.015
	CON	136 ± 20	130 ± 20			0.043
FD/ED	PT	110 ± 31	127 ± 20	0.776	0.256	0.255
	BT	102 ± 24	116 ± 19			0.193
	CON	102 ± 35	126 ± 26 *			0.393
RF/BF	PT	119 ± 21	85 ± 13 *	0.031	3.803	0.764
	BT	110 ± 32	95 ± 25			0.206
	CON	113 ± 27	91 ± 24 *			0.410

RMS: root mean square; EMG: electromyography; DA: deltoid anterior; DP: deltoid posterior; BB: biceps brachii; TB: triceps brachii; FD: flexor digitorum; ED: extensor digitorum; RF: rectus femoris; BF: biceps femoris; * *p* < 0.05 vs. Pre.

## Data Availability

Data supporting the reported results can be found in the study by Zaferanieh et al. [23].
